# Del11q-positive CLL lymphocytes exhibit altered glutamine metabolism and differential response to GLS1 and glucose metabolism inhibition

**DOI:** 10.1038/s41408-017-0039-2

**Published:** 2018-01-24

**Authors:** Gabriela Galicia-Vázquez, Sarah Smith, Raquel Aloyz

**Affiliations:** 1 Lady Davis Institute for Medical Research, Jewish General Hospital, Montreal, Quebec Canada; 2 Segal Cancer Center, Jewish General Hospital, Montreal, Quebec Canada; 3 Division of Experimental Medicine, McGill University, Montreal, Quebec Canada; 4 Department of Oncology, McGill University, Montreal, Quebec Canada

Chronic lymphocytic leukemia (CLL) is characterized by the clonal expansion of malignant B cells, and their accumulation in the blood stream and homing tissues. In the CLL context, the chromosomal aberrations del17p and del11q, spanning TP53 and ATM locus, respectively, are considered poor outcome predictors^1,2^. As well, CD38, ZAP70, and unmutated status of the IgVH locus represent bad prognosis markers^1^. Despite the outstanding clinical results yielded by ibrutinib (mainly used in relapsed or refractory settings), no complete remission is guaranteed due to the arising of resistance and relapse upon treatment discontinuation. The lack of tailored therapies upon ibrutinib failure represents a major challenge, particularly in del11q and del17p cases refractory to conventional therapies^3^. Recently, the analysis of metabolic reprogramming has uncovered tumor cell vulnerabilities, leading to the development of novel therapeutic approaches; such as the use of ritonavir (glucose uptake inhibitor) and metformin (OxPhos inhibitor) for multiple myeloma treatment^4^.

The aim of this work was to define targetable metabolic features in CLL lymphocytes with respect to their del11q status, since *ATM*—a reactive oxygen species (ROS) sensor and metabolic regulator^5^—and *miR125*—metabolic regulator^6^—genes are comprised within del11q. Also, del11q has been linked to increased insulin receptor expression^7^. We used a panel of 26 CLL primary samples donated from affected patients, 23% of which were del11q-positive, which represent occurrence of del11q cases in the clinical setting (~20%), while del17p cases were excluded (Supplementary Table 1). CLL lymphocytes were maintained in a physiological glucose concentration, since our group noticed that CLL cells exposed to high or limited glucose levels display different metabolic responses^8^. Under basal conditions, no significant difference was found in glucose or glutamine uptake, total or reduced glutathione, and ROS levels (Supplementary Fig. 1a–d) between del11q-positive (hereafter del11q) and del11q-negative (hereafter wild-type) CLL lymphocytes, suggesting that del11q CLL lymphocytes are adapted to reduced ATM expression.

CLL lymphocyte metabolism was explored by challenging these lymphocytes with a panel of metabolic inhibitors (Supplementary Table 2). The disturbance of glycolysis (2DG), glucose uptake (ritonavir) or OxPhos (oligomycin) decreased all CLL cell viability. Del11q CLL cells displayed enhanced sensitivity for the three treatments. Inhibiting fatty acid oxidation (etomoxir) was equally cytotoxic to all CLL lymphocytes; conversely, the inhibition of the pentose phosphate pathway (PPP) (DHEA) or the one-carbon metabolism (AMPA) was not cytotoxic to CLL cells (Fig. 1a).

**Fig. 1:**
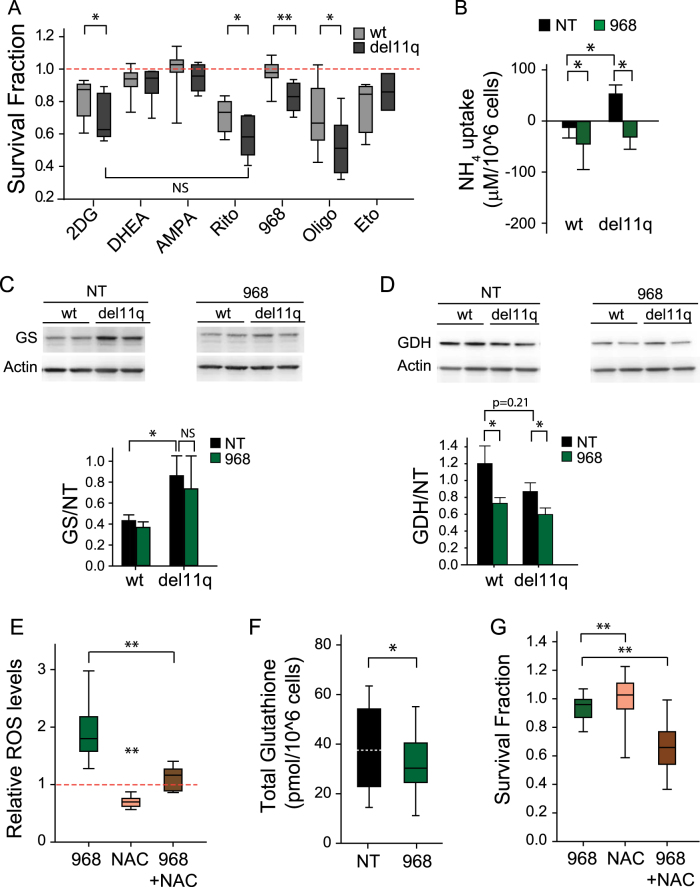
Del11q CLL lymphocytes display glutamine metabolic alterations. **a**, **g** Survival fraction (relative to non-treated control (NT)) of CLL lymphocytes treated with metabolic inhibitors for 48 h. **a** *n* = 23, **g** *n* = 19. **b** Ammonia uptake after 24 h compound 968 treatment (*n* = 14). **c** GS (*n* = 13) and **d** GDH (*n* = 13). Western blot of samples treated with compound 968 for 24 h. Representative images (upper panel) and quantification (lower panel) are shown. **e** Relative ROS values after 24 h treatment (*n* = 19, *n* (968 + NAC) = 8). **f** Population median value of total glutathione after 24 h of compound 968 treatment (*n* = 12). NS, not significant, *p < 0.05, **p < 0.001

Curtailing the first step of glutamine metabolism, via glutaminase (GLS1) inhibition (compound 968), was cytotoxic only to del11q CLL cells (Fig. 1a). Since glutamine and glutamate uptake were similar between del11q and wild-type CLL cells (Supplementary Fig. 1e, f), it is likely that differential utilization of these metabolites is associated with del11q status. A distinctive characteristic of del11q CLL cells was the consumption of ammonia under basal conditions, while wild-type CLL lymphocytes secrete ammonia (Fig. 1b). This suggests enhanced amino-acid catabolism in wild-type CLL cells. Accordingly, glutamine synthetase (GS) expression was higher in del11q compared to wild-type CLL lymphocytes, while glutamate dehydrogenase (GDH) expression followed the opposite trend (Fig. 1c, d). The former suggests that glutamine is synthesized de novo via GS and the latter that transaminase reactions using α-ketoglutarate for glutamate synthesis is favored in del11q CLL cells. As well, reduced oxidative deamination of glutamate via GDH could account for decreased extracellular ammonia accumulation in del11q CLL lymphocytes. Glutamine synthesis is favored when ammonia detoxification is required, and for tricarboxylic acid cycle cataplerosis in rich nutrient conditions^9^; however, glutamine uptake and GLS1 expression were similar between subsets (Supplementary Fig. 1e, g). The latter raises the possibility that differences in energetic or biosynthetic requirements exist between wild-type and del11q CLL lymphocytes. GLS1 inhibition increased extracellular ammonia in del11q CLL lymphocytes (Fig. 1b). However, glutamine and glutamate uptake were not affected by compound 968, suggesting that CLL lymphocytes are poised to maintain constant extracellular concentrations of these metabolites (Supplementary Fig. 1e, f). Our findings suggest that amino-acid catabolism contributes to glutamate production in CLL lymphocytes. Also, we propose that GDH contribution to glutamate pools is limited under GLS1 inhibition, since GDH expression was significantly reduced after compound 968 treatment, regardless of del11q status (Fig. 1d).

ROS levels increased upon GLS1 inhibition, a possible consequence of decreased total glutathione—a tri-peptide formed by glutamate, glycine, and cysteine (Fig. 1e, f). Noteworthy, the cysteine precursor N-Acetyl-L-cysteine (NAC) reduced ROS levels without affecting survival (Fig. 1e, g), and promoted the accumulation of extracellular glutamate (Supplementary Fig. 2a). Since CLL lymphocytes express the cysteine/glutamate xCT antiporter, we propose that NAC uptake limits glutamate-dependent processes and reduces nutrient usage plasticity in CLL lymphocytes, as recently reported for other cancer cells^10^. Accordingly, NAC enhanced compound 968 cytotoxicity regardless of del11q status (Fig. 1g), which could not be compensated by increased glutamine uptake (Supplementary Fig. 2b). Furthermore, compound 968 triggered an increase in glucose uptake in wild-type CLL lymphocytes only (Supplementary Fig. 2c). Based on our results, we infer that wild-type cells but not del11q lymphocytes can efficiently rewire their amino-acid and glucose metabolism to compensate GLS1 inhibition.

To further explore differences in glucose metabolism by del11q, we made drug combination treatments with 2DG and DHEA. Although PPP inhibition was not cytotoxic to CLL lymphocytes, in the context of glycolysis inhibition, DHEA potentiated 2DG cytotoxicity (Fig. 2a, Combination Index = 0.67), especially in del11q CLL clones. Such an effect has been previously reported for other cancer cell lines^11^, and is suggestive of the dependence of CLL lymphocytes on the coordinated use of glycolysis and PPP to survive. In line with differential glucose metabolic reprogramming, 2DG decreased glucose uptake only in del11q CLL cells (Fig. 2b), suggesting increased dependency on glycolysis for survival. Contrariwise, DHEA increased glucose uptake and metabolic activity (detected by C-12 Resazurin reduction) only in wild-type CLL clones (Fig. 2b and Supplementary Fig. 3a), suggesting that glucose flux through the PPP is favored in this subset, and that DHEA triggers the redirection of glucose carbons to glycolysis, promoting mitochondrial activity. The shunting of glucose from glycolysis to PPP was previously documented in red blood cells under 2DG treatment and it is believed to occur in quiescence in response to high NADPH demands^12,13^. However, 2DG plus DHEA-induced cytotoxicity is not driven by oxidative stress, since the drugs (alone or in combination) did not significantly affect ROS levels in the CLL clones tested (Supplementary Fig. 3b). Of note, similar cytotoxicity was obtained upon 2DG and ritonavir treatment in del11q CLL lymphocytes, making it possible that GLUT4 is their main glucose transporter (Fig. 1a). Unexpectedly, AMPA antagonized 2DG-induced cytotoxicity regardless of del11q status (Fig. 2c). It is possible that one-carbon metabolism drives glucose-derived carbons out of glycolysis in basal conditions, and AMPA treatment allows these carbons back to glycolysis. In this scenario, serine accumulation upon SHMT1/2 inhibition could contribute to PKM2 activation, enhancing glycolysis flux, downstream of hexokinase.

**Fig. 2:**
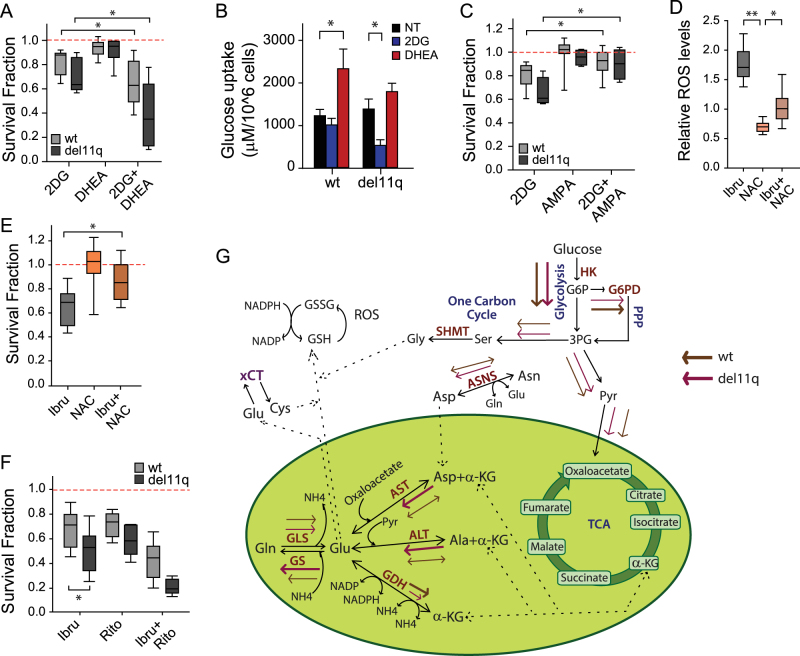
Effect of glucose metabolic inhibitors and ibrutinib on CLL metabolism. **a**, **c**, **e**, **f** Survival fraction (relative to non-treated control (NT)) of CLL lymphocytes treated with metabolic inhibitors for 48 h. **b** *n* = 21, **c** *n* = 12, **e** *n* = 19, **f** *n* = 23. **b** Glucose uptake after 24 h compound treatment (*n* = 15). **d** Relative ROS median values of CLL cells after 24 h of ibrutinib and/or NAC treatment (*n* = 20). *p < 0.05, **p < 0.001. **g** Model for basal wild-type and del11q CLL cell metabolism. The proposed metabolic flux in CLL lymphocytes is indicated with brown (wild-type) and pink (del11q) arrows. Del11q CLL lymphocytes promote glutamine synthesis, while decreasing GDH reaction. Glutamate production might be maintained via amino-acid catabolism through transaminase reactions, while glycolysis could provide the necessary metabolites to feed the TCA, PPP, and one-carbon metabolism. Wild-type CLL lymphocytes have a significant flux of glucose-derived carbons through PPP, while their amino-acid metabolism tends to generate ammonia

Recently, it was reported that enhanced OxPhos is associated with unmutated IgVH and advanced Rai stage, but not with del11q or del17p in CLL lymphocytes (on hyperglycemic conditions). Furthermore, in contrast to our results below using ibrutinib, pharmacological targeting of the PI3K pathway in primary CLL lymphocytes decreased OxPhos without affecting glutamine/glucose uptake or ROS levels^14^.

Bruton's Tyrosine Kinase (BTK) influence on metabolism was assessed by ibrutinib treatment. Ibrutinib increased glucose uptake, glutamine uptake, and ammonia secretion, without disturbing glutamate secretion (Supplementary Fig. 4a–d), regardless of del11q status. In line with enhanced ROS levels upon ibrutinib treatment (Fig. 2d), total and reduced glutathione levels were decreased by 60% and 90%, respectively, as well as NADP/NADPH ratio (Supplementary Fig. 4e–g). Ibrutinib-induced cytotoxicity was influenced by oxidative stress, since NAC could decrease ROS levels along with ibrutinib-induced cytotoxicity in CLL lymphocytes (Fig. 2d, e). ROS levels increased upon GLS1or glutamine deprivation (Supplementary Fig. 4h), suggesting that the contribution of glutamine metabolism to ROS control is not compensated by other NADPH-generating pathways, and that ibrutinib affects glutamine metabolism by reducing glutamine pools. This is supported by the observation of decreased extracellular accumulation of glutamate upon GLS1 inhibition in ibrutinib treated CLL lymphocytes (Supplementary Fig. 4d). Importantly, ibrutinib-induced cytotoxicity was higher on del11q CLL lymphocytes (Fig. 2f), in line with increased sensitivity to GLS1 inhibition. Noteworthy, the simultaneous inhibition of BTK and glucose uptake was highly cytotoxic to CLL lymphocytes, especially to del11q CLL cells, underscoring the role of BTK in metabolic homeostasis (Fig. 2f). We presume that in addition to its effects on BCR signaling, ibrutinib disturbs glutamine metabolism possibly by decreasing glutamine/glutamate availability.

Based on our results, we propose that the differences in glutamine metabolism displayed by del11q CLL lymphocytes could account for their oversensitivity to metabolic stress, due to reduced metabolic plasticity (Fig. 2g). This study opens the door for the development of personalized medicine for CLL del11q-positive patients, especially in cases presenting relapse to chemotherapy or ibrutinib treatment. Moreover, the availability of GLS1 inhibitors such as CB-839—currently in clinical trials for AML and ALL (clinicaltrials.gov)—paves the way for the targeting of glutamine metabolism in CLL.

## Electronic supplementary material

Supplementary Information

Supplementary Figure 1

Supplementary Figure 2

Supplementary Figure 3

Supplementary Figure 4

Supplementary Table 1

Supplementary Table 2
