# Supplementary material for: Del11q-positive CLL lymphocytes exhibit altered glutamine metabolism and differential response to GLS1 and glucose metabolism inhibition
Source: Blood Cancer J. 2018 Jan 24;8(1):13. doi: 10.1038/s41408-017-0039-2 (PMC5802573; doi:10.1038/s41408-017-0039-2)
Supplement: Supplementary file 1 — Supplementary Information [file 41408_2017_39_MOESM1_ESM.docx]

**Supplementary Information**

**MATERIALS AND METHODS**

**Patient samples.** We used 26 patient B-CLL cell samples from our CLL primary cell bank. Samples were obtained with the patients’ informed consent, in accordance with the ethical approval granted by the Jewish General Hospital Research Ethics Committee. *The characteristics of these samples are summarized on Supplementary Table 1.*

**Cell Culture, and compound treatments.** Primary CLL cells were thawed and cultured overnight in AIM-V (GIBCO, 12055-091), 10% FBS at 37°C. Then, cells were seeded, treated and incubated at 37°C and 5% CO_2_ with the compounds *described in Supplementary Table 2*: Cells were treated for 24 or 48h with the following compounds: Ibrutinib (Selleckchem, S2680), Oligomycin A (Sigma, 75351), 2-Deoxy-D-glucose (Sigma, D6134), Ritonavir (Selleckchem, S1185), Compound 968 (Calbiochem, 352010), DHEA (Sigma, D-063), AMPA (Sigma, 324817), Etomoxir (Sigma, E1905). Rescue experiments were done with: 4 mM N-Acetyl-L-cysteine (NAC) (Sigma). Physiological 5.5 mM glucose RPMI is a 1:1 mix of RPMI 1640 (Wisent, 350-000, 11 mM glucose) and RPMI 1640 w/o glucose (GIBCO, 11879-020). Glutamine free RPMI media is a 1:1 mix of RPMI 1640 w/o glucose w/o glutamine (Wisent, 350-061) and RPMI 1640 w/o glutamine (Wisent, 350-030). All RPMI media were supplemented with 10% FBS, 25 mM HEPES, and 100 U/mL penicillin/streptomycin.

**Viability/ Metabolic activity Assay.** The Metabolic Activity/Annexin V/Dead Cell Apoptosis Kit with C12 Resazurin, APC annexin V, and SYTOX® Green for Flow Cytometry (Invitrogen, V35114) was used following manufacturer instructions. Data was collected by flow cytometry (BD FACSAria Fusion cell sorter).

**Glucose, glutamine, glutamate, and ammonia quantification.** Cell media was collected for metabolite quantification in a NOVA Bioprofile Analyzer 400 at GCRC Metabolomics Core (McGill University). Glucose, glutamine, glutamate, and ammonia uptake were calculated by comparing the change in concentration between media collected after treatment and unused media (blank), metabolite use was normalized to cell number (µM/10^6 cells).

**ROS determination.** CellROX® Green Flow Cytometry Assay Kit (Invitrogen, C10492) was used as directed by the manufacturer. Data was collected by flow cytometry (BD FACSAria Fusion cell sorter).

**Glutathione concentration determination.** Oxidized and Reduced glutathione were determined with GSH/GSSG-Glo Assay (Promega, V6611), according to manufacturer instructions. After 24h treatment, 1.5x10^6^ cells per sample was analyzed. A glutathione standard curve was done to calculate glutathione concentration in the samples. Measurements were obtained using a FluoStar Optima Reader (BMG Labtech).

**NADP/NADPH ratio determination.** Oxidized and Reduced NADP were determined with NADP/NADPH-Glo Assay (Promega, G9081), according to manufacturer instructions. After 24h treatment, 1.2x10^6^ cells per sample was analyzed. Measurements were obtained using a FluoStar Optima Reader (BMG Labtech).

**Immunoblotting Analysis.** Cell pellets were collected 24 h after treatment, protein samples were fractionated on 4–12% Bis-Tris gels, transferred to nitrocellulose membranes, and probed against: GDH (Cell Signaling, cs12793), GS (BD Biosciences, 610517), p-AMPK (Cell Signaling, cs2535), β-Actin (Santa Cruz, sc-1616). GS and GDH non-treated samples were loaded in the same gel with their paired compound 968 treated samples.

**Data Analysis.** Flow cytometry data was analyzed using FlowJo V.10 software. Western Blot band quantification was performed using ImageJ 1.49v software (NIH, USA). Statistical analysis was performed using Sigma Plot version 13.0 (Systat Software Inc.). Two tailed t-tests were used to compare two groups. Differences were considered significant if p value was <0.05. Combination Indexes (CI) were calculated with Highest Single Agent method (reviewed in Foucquier and Guedj, PRP, 2015). Bar plots represent the Mean +/- SEM. The center value in the Box plots represents the median, the boundaries of the box show the 25^th^ and 75^th^ percentiles, while the error bars indicate the 90^th^ and 10^th^ percentiles.

**SUPPLEMENTARY FIGURE LEGENDS**

**Supplementary Figure 1. Impact of del11q on basal CLL metabolism and under GLS1 inhibition. A.** Basal Glucose (n=15), and glutamine (n=15) uptake after 24h of compound treatment**. B.** Population median value of total glutathione present in NT cells (n=12). **C.** Reduced/Oxidized glutathione ratio in NT cells (n=12). **D.** Population median value of CellRox assays performed in NT cells (n=26). **E.** Glutamine (n=15) **F.** Glutamate (n=15) uptake after 24h of compound 968 treatment. **G.** GLS1 basal expression. Representative western blot images (upper panel) and quantification (lower panel) are shown (n=20). NS-Not significant.

**Supplementary Figure 2. Effect of cysteine intake and GLS1 inhibition in extracellular metabolite concentration. A.** Glutamate (n=6) **B.** Glutamine (n=6) uptake after 24h of NAC treatment. **C.** Glucose (n=14) uptake after 24h compound 968 treatment. *p<0.05.

**Supplementary Figure 3. Cytotoxicity induced by glucose metabolism inhibition is not caused by oxidative stress. A.** Relative metabolic activity after 48h compound treatment (n=24). **B.** Relative ROS values after 24h compound treatment (n=21). *p<0.05, **p<0.001.

**Supplementary Figure 4. Ibrutinib modifies CLL metabolism. A.** Glucose (n=21) **B.** Glutamine (n=20) **C.** Ammonia (n=21) and **D.** Glutamate (n=20) uptake after 24h of ibrutinib treatment. **E.** Relative glutathione (pmol per million cells) present CLL cells after 24h of ibrutinib treatment (n=12). **F.** Reduced/Oxidized glutathione ratio after 24h of ibrutinib treatment (n=12) **G.** NADP/NADPH ratio after 24h of ibrutinib treatment (n=5). **H.** Relative ROS median values of CLL cells after 24h of glutamine deprivation (n=26). *p<0.05, **p<0.001
